# Deep learning-based ultrasound transducer induced CT metal artifact reduction using generative adversarial networks for ultrasound-guided cardiac radioablation

**DOI:** 10.1007/s13246-023-01307-7

**Published:** 2023-08-07

**Authors:** Sathyathas Puvanasunthararajah, Saskia M. Camps, Marie-Luise Wille, Davide Fontanarosa

**Affiliations:** 1 School of Clinical Sciences, Queensland University of Technology, Brisbane, QLD Australia; 2 Centre for Biomedical Technologies, Queensland University of Technology, Brisbane, QLD Australia; 3 EBAMed SA, Geneva, Switzerland; 4 School of Mechanical, Medical & Process Engineering, Faculty of Engineering, Queensland University of Technology, Brisbane, QLD Australia; 5 ARC ITTC for Multiscale 3D Imaging, Modelling, and Manufacturing, Queensland University of Technology, Brisbane, QLD Australia

**Keywords:** Cardiac radioablation, Ultrasound guidance, Metal artifact reduction, Deep learning, Computed tomography

## Abstract

In US-guided cardiac radioablation, a possible workflow includes simultaneous US and planning CT acquisitions, which can result in US transducer-induced metal artifacts on the planning CT scans. To reduce the impact of these artifacts, a metal artifact reduction (MAR) algorithm has been developed based on a deep learning Generative Adversarial Network called Cycle-MAR, and compared with iMAR (Siemens), O-MAR (Philips) and MDT (ReVision Radiology), and CCS-MAR (Combined Clustered Scan-based MAR). Cycle-MAR was trained with a supervised learning scheme using sets of paired clinical CT scans with and without simulated artifacts. It was then evaluated on CT scans with real artifacts of an anthropomorphic phantom, and on sets of clinical CT scans with simulated artifacts which were not used for Cycle-MAR training. Image quality metrics and HU value-based analysis were used to evaluate the performance of Cycle-MAR compared to the other algorithms. The proposed Cycle-MAR network effectively reduces the negative impact of the metal artifacts. For example, the calculated HU value improvement percentage for the cardiac structures in the clinical CT scans was 59.58%, 62.22%, and 72.84% after MDT, CCS-MAR, and Cycle-MAR application, respectively. The application of MAR algorithms reduces the impact of US transducer-induced metal artifacts on CT scans. In comparison to iMAR, O-MAR, MDT, and CCS-MAR, the application of developed Cycle-MAR network on CT scans performs better in reducing these metal artifacts.

## Introduction

Cardiac radioablation is a new non-invasive modality for the treatment of cardiac arrhythmias. This treatment method is based on delivering a radiation dose to the arrhythmogenic tissues using external beam radiation therapy [1–3]. A typical treatment workflow includes acquiring a planning computed tomography (CT) used to delineate the arrhythmogenic tissue (target) and the organs-at-risk (OARs) for the Hounsfield Units (HU) derived foreseen radiation dose calculation. At treatment, the dose is then delivered to the target while sparing the OARs as much as possible. However, the complex cardiorespiratory motion may impact the accuracy of dose delivery [4, 5]. This makes real-time monitoring of the cardiorespiratory motion of paramount importance to achieve safe and effective treatment delivery.

A possible candidate image modality for real-time guidance in cardiac radioablation is transthoracic ultrasound (US) imaging [6, 7]. This approach relies on identifying the cardiac tissue position using US imaging at both the simulation and the treatment delivery stages. Comparing the two allows to compensate for possible displacements of cardiac structures at treatment. To minimize clinical workflow steps and ease treatment planning, it may be favourable to acquire the US scans simultaneously with the planning CT scan at the simulation stage. However, this approach is prone to creating transducer-induced metal artifacts on the CT scans, caused by the internal metal components of the US transducers [8, 9].

Metal artifacts may generate improper representations of anatomical structures and incorrect HU values, resulting in potentially inaccurate radiation dose calculation [10–12]. Several algorithms for metal artifact reduction (MAR) have been developed, mainly focusing on the artifacts generated by implanted metal structures. The majority of these MAR algorithms, both commercially available and research-based, follow a conventional analytical approach. Recently, new works based on deep learning have been proposed [11, 12]. Commercially available MAR algorithms use an iterative approach of correction of CT projection data [12]. Among them, the Orthopaedics Metal Artifact Reduction (O-MAR, Philips Health System) and the iterative Metal Artifact Reduction (iMAR, Siemens Healthcare) algorithms were widely investigated for the improvement of radiation therapy planning [13–21]. Among the research-based MAR algorithms, Metal Deletion Technique (MDT, ReVision Radiology) [22] was the one most often compared to the commercially available algorithms [23–26]. All these algorithms typically suffer from improper restoration of HU values, from distortion of anatomical structures, and even from creation of secondary artifacts [11]. Recently, our group [27] investigated a newly developed MAR algorithm, Combined Clustered Scan-based MAR (CCS-MAR), which followed the traditional analytical approach, and compared its performance to commonly used commercial and research-based MAR algorithms. The results of this study revealed that further development or improvements were needed to reduce residual artifacts and further improve HU value restoration capabilities.

In recent years, the development of deep learning-based algorithms for metal artifact reduction in CT imaging gained significant interest [28–38]. In general, to learn in a supervised manner [39, 40] the complex metal artifact patterns and propagation, these algorithms require data including CT scans with artifacts (CT_art_), and corresponding artifact-free (CT_ref_) scans. In the absence of adequate paired data, typical metal artifacts which resemble real clinical scenarios can be simulated [28, 30, 31, 38]. For our work, we have used generative adversarial networks (GAN) [41]. Typically, these architectures consist of a generator and a discriminator, which are types of convolutional neural networks (CNN) [41, 42]. The generator and the discriminator are trained in an adversarial manner to perform the transformation. We have focused in particular on their extension CycleGAN [43], which utilizes two GAN architectures, and has already been studied in literature for the transformation of CT_art_ scans into artifact-reduced CT (CT_cor_) scans for radiation therapy applications [28, 29, 31].

This work aims to develop a deep learning-based MAR algorithm for the reduction of US transducer-induced metal artifacts on CT scans. The proposed algorithm was designed following a supervised learning scheme with a CycleGAN architecture using paired clinical CT scans. Then it was evaluated on phantom CT scans with real artifacts and clinical CT scans with simulated artifacts. In addition, the performance of CycleGAN has been compared with the performance of iMAR, O-MAR, MDT and CCS-MAR.

## Materials and methods

### CT data preparation

#### Clinical CT scans

Paired clinical CT scans with and without US transducer-induced metal artifacts were used. In particular, DICOM CT scans of the thoracic region were utilized from the “COVID19-CT-Dataset” online database [44]. Initially, CT scans from 180 patients were downloaded and visually inspected for suitability. CT scans or CT slices with large COVID19 induced density changes in the lungs region, with suboptimal quality due to low resolution, and/or with presence of metal artifacts resulting from external foreign bodies were excluded. This resulted in CT scans from 84 patients, and from each of these CT scans, axial CT slices composed of 22–100 slices per patient covering the cardiac structure from the apex to the base were selected. These CT scans consisted of 512 × 512-pixel slices with 1.5 or 3 mm slice thickness [45].

**Fig. 1:**
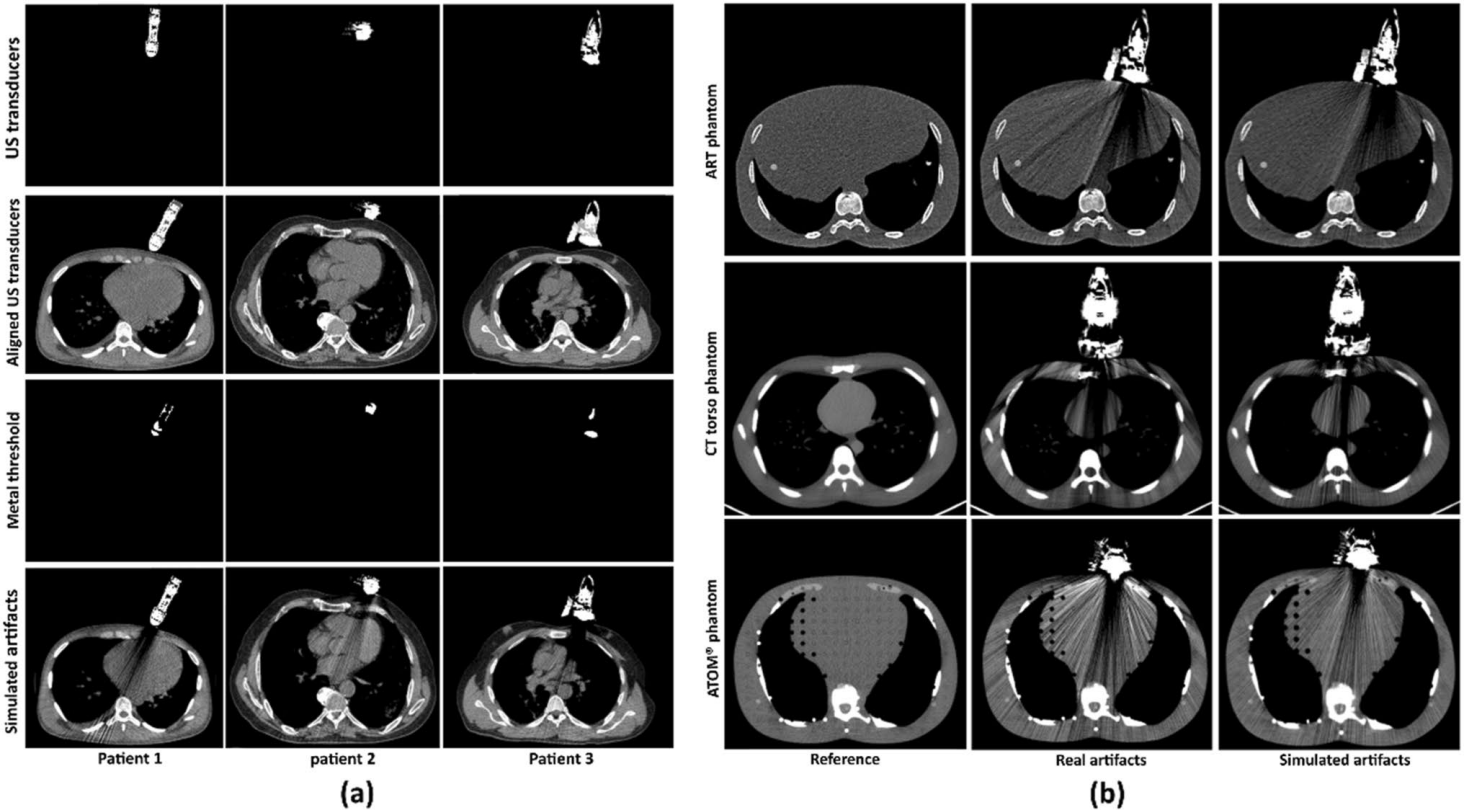
**a** The procedure for simulation of US transducer-induced metal artifact on clinical CT scans. The first and second row show the segmented US transducers from the phantom CT scans and the aligned US transducers with the suitable clinical CT slices, respectively. Pixels containing metal parts of a US transducer and the CT slices consisting of simulated artifacts are shown in the last two rows, respectively. **b** Visual representation of the real artifacts and the simulated artifacts on the phantom scans. [Window level/ width: 50/ 350]

To simulate the metal artifacts on the CT scans, Sakamoto et al. [46]. developed a MatLab package based on the study by Zhang et al. [38]. This package was modified in our work for the specific simulation of US transducer-induced metal artifacts on the selected COVID19-CT-Dataset (See Fig. 1 a). In our procedure, initially the pixels identifying the US transducers on the phantom CT_art_ scans were manually segmented and stored separately. Then, the segmented US transducers were copied to the clinical CT scans and positioned on the scans using rigid rotation-translations to be imaging the cardiac structures. A threshold value of 2000 HU was applied on the imported US transducer to extract metal components, which were then saved as binary images. The metal extraction threshold value of 2000 HU was chosen according to the previous research published in the literature [27, 47, 48]. Then, pre-defined HU thresholds were used to segment the bone, lung, and water-equivalent tissues on the clinical CT_ref_ slices. To convert the HU values of the pixels into linear attenuation coefficient for varying X-ray energies, corresponding mass attenuation coefficients were used from the NIST [49] database. Subsequently, the polychromatic projection data for corresponding X-ray energies were simulated from the segmented bone, lung, water equivalent tissue and from the metal binary image. As the metal components of a US transducer consist of lead, zirconate, and titanate [50], the average mass attenuation coefficient value of these metals was used to generate the projection data. Consequently, metal-containing projection data was created from those simulated projections with Poisson distribution for the reconstruction of a CT_art_ slice.

To check the correctness of this artifact simulation method, phantom CT scans were utilized (See Fig. 1 b). From a particular phantom CT scan, initially, CT_ref_ and the corresponding CT_art_ slices were selected. Then, the US transducer-induced metal artifacts were simulated on the phantom CT_ref_ slices based on the procedure described above. The simulated CT_art_ slices were visually validated against the corresponding phantom CT_art_ slices comparing them to the real US transducer-induced metal artifacts.

#### Phantom CT scans

**Fig. 2:**
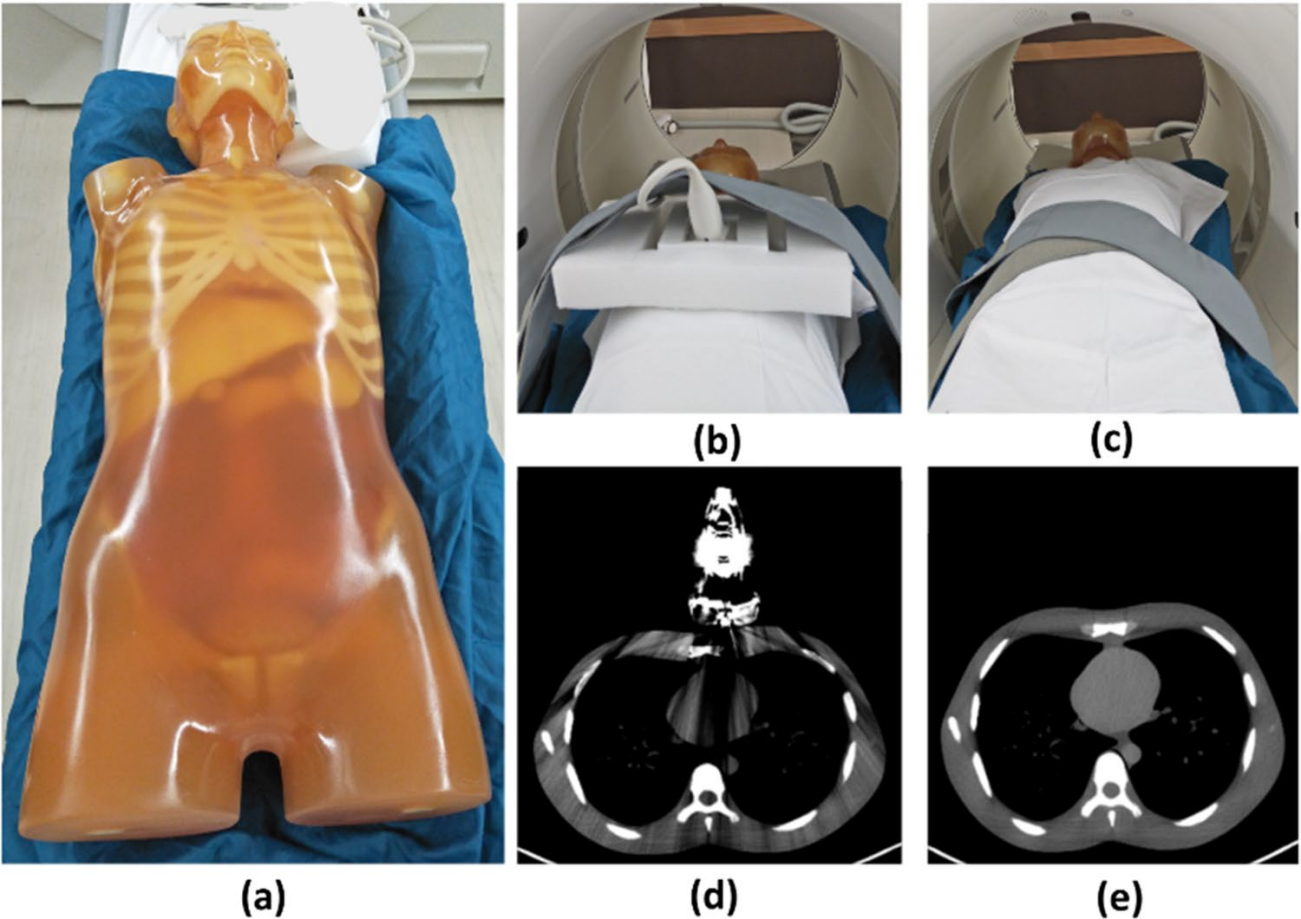
Workflow to obtain the dataset pairs: in (**a**) the CT torso phantom is shown. The positioning of the phantom with (**b**) and without (**c**) the linear volume array transducer resulted in CT scans as shown in (**d**) and (**e**)

Table 1 shows the combinations of CT scanners, anthropomorphic phantoms, and US transducers used in this work. In particular, three types of adult anthropomorphic phantoms were used to scan with and without a total of four types of US transducers. The utilized anthropomorphic phantoms were an ART-211 male phantom (ART, Radiology Support Devices, Long Beach, CA, USA); an ATOM® male phantom (CIRS, Model-701, Norfolk, VA, USA); and a CT torso phantom (CT Torso, Model CTU-41, Kyoto Kagaku Ltd, Japan). These anthropomorphic phantoms were constructed using tissue-equivalent epoxy materials that mimics the density and attenuation characteristics of human tissues. They include a range of components, such as cardiac structures, air-equivalent materials for simulating lungs, and bone-like materials with simulated air pockets. To obtain the paired CT scans, each phantom was CT scanned with and without a US transducer, resulting in a CT_art_ scan and the corresponding CT_ref_ scan, respectively (See Fig. 2 for an example of the procedure). The US transducers were positioned on the phantoms at various angles to be suitable for proper imaging of the heart. As the dimensions of US transducers, including the size and width of their metal components, can have an impact on the creation of metal artifacts on CT scans. Among the US transducers used, the linear volume array transducer was the largest and had the widest metal component, which was measured to be 6 cm using CT scans, and induced a greater quantity of metal artifacts compared to the other transducers.

**Table 1:** The details of utilized CT scanners, anthropomorphic phantoms, and the US transducers

CT scanner (Model)	Clinical site	Phantom	US transducers
Siemens CT (SOMATOM Definition AS)	Kantonnspital Aarau, Aarau, Switzerland	ART-211	Single-plane phased array, Bi-plane phased array
Philips CT (Brilliance Big Bore)	Geneva University Hospital, Geneva, Switzerland	ATOM®	Single-plane phased array, Bi-plane phased array
Siemens PET-CT (Biograph 128)	Herston Imaging Research Facility (HIRF), Brisbane, Australia	CT torso	Linear array, Linear volume array

Single-plane phased array: Telemed P5-1S15-A6 from Telemed (UAB, Vilnius, Lithuania); bi-plane phased array: Terason XY mini from Teratech Corporation (Burlington, MA, USA); linear array: Philips L7-4 Philips Healthcare (Andover, MA, USA) and linear volumetric array: Philips VL13-5

### Cycle-MAR network

**Fig. 3:**
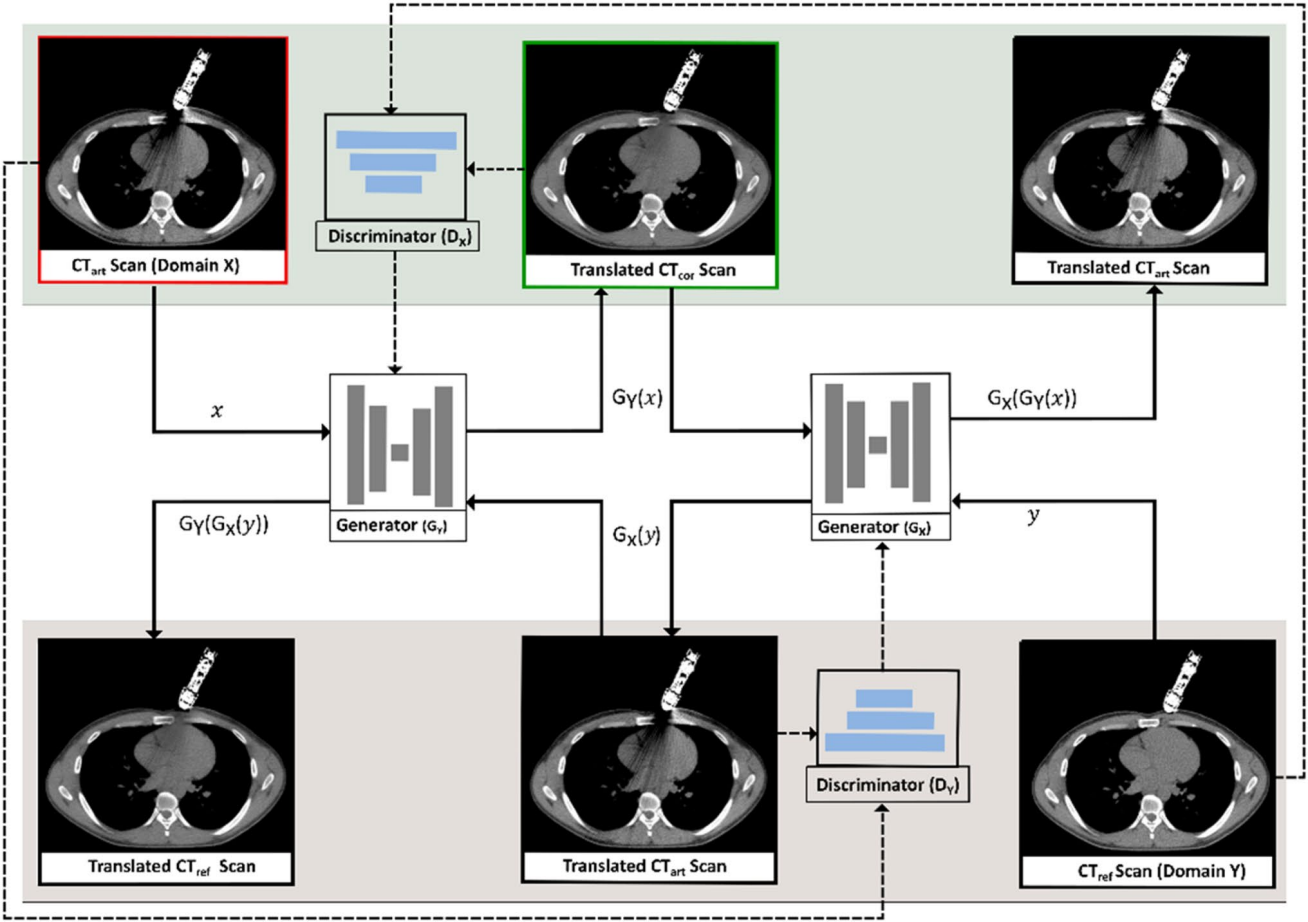
Training workflow of the Cycle-MAR network for the reduction of the US transducer-induced metal artifacts. It has two mapping functions: generator (G_Y_) transforms the CT_art_ scan (domain X) into CT_cor_ scan, while generator (G_X_) transforms the CT_ref_ scan (domain Y) into an CT_art_ scan. Discriminator (D_X_) and discriminator (D_Y_) aim to distinguish translated CT_ref_ scan from the CT_ref_ (domain Y) and distinguish the translated CT_art_ scan from the CT_art_ scan (domain X), respectively

The CycleGAN model [43] has been proposed in literature for unpaired training data. However, we used paired data in this work to enforce the restoration accuracy of anatomical structures and HU values [51]. The workflow of the developed Cycle-MAR network is illustrated in Fig. 3.

The CycleGAN translates the metal artifact domain (X) into an artifact-free domain (Y) by using adversarial loss (${\mathcal{L}}_{adv})$, cycle consistency loss ${\mathcal{L}}_{\left(cycle\right)}$, and identity loss ${\mathcal{L}}_{\left(identity\right)}$. This process includes two mapping functions, ${G}_{X}:X\to Y$ and ${G}_{Y}:Y\to X$. The mapping function ${G}_{X}$ translates a CT_art_ slice into a CT_cor_ slice, whereas ${G}_{Y}$ translates a CT_ref_ slice into CT_art_ slice. The network also consists of two adversarial discriminators ${D}_{X}$ and ${D}_{Y}$ which aim to distinguish the translated domain as fake. ${D}_{X}$ aims to distinguish between $x$ from ${G}_{X}\left(y\right)$ and ${D}_{Y}$ aims to distinguish $y$ from ${G}_{Y}\left(x\right)$. For ${G}_{Y}$, the (${\mathcal{L}}_{adv})$ is the mean squared error (MSE) between output ${G}_{Y}\left(x\right)$ and target domain $Y$. The ${\mathcal{L}}_{\left(cycle\right)}$ calculates the translation error between $x$and ${G}_{X}\left({G}_{Y}\left(x\right)\right)$ through the translation of $X\to Y \to X$ and in vice versa of $Y\to X \to Y$. The ${\mathcal{L}}_{\left(identity\right)}$ was introduced to regularize the ${G}_{X}$ and ${G}_{Y}$ to not induce any changes when x and y were the input for them, respectively.

In this study, regularization parameters values of ${\lambda }_{cycle}=10$ for, and${\lambda }_{identity}=15$ were chosen among several examined parameter sets. To implement Cycle-MAR, the ResNet [52] and the PatchGAN [42] architectures were used as the generator and the discriminator, respectively. The network was trained using the Adam optimizer [53] for 500 epochs using a batch size of 1 which produced the best results and validated with five-fold cross-validation. The cross-validation with five-fold was chosen based on the previous studies [29, 33, 54]. To avoid overfitting, data augmentation methods of rotation, horizontal flip, resized crop and image perspective were utilized during the training session. The Cycle-MAR network was implemented using Python 3.8.8, with the PyTorch (version 1.10.2) framework. A NVIDIA (TITAN RTX) GPU with 25GB was used throughout the experiments.

### Data split for training and testing

Table 2 shows the data split strategy for the training and testing of Cycle-MAR. The network was trained using randomly selected paired clinical CT scans consisting of CT_ref_ scans and the corresponding simulated CT_art_ scans. Then it was tested on the phantom CT scans with real artifacts, and on the clinical CT scans with simulated artifacts. The HU values on the clinical CT scans were clipped between − 1000 and 1000, and the remaining pixel values were normalized between − 1 and 1 to improve the training efficiency [28].

**Table 2:** Data split strategy for training and testing of the Cycle-MAR network

Data	Data split	Patient/phantom	Number of CT slices (%)
Clinical CT scans	Training (paired CT_ref_ & CT_art_)	70	3515 (85)
	Testing (CT_art_)	14	650 (15)
Phantom CT scans	Testing (CT_art_)	ART	20 (41.1)
		ATOM®	23 (31.5)
		CT torso	30 (27.4)

### Comparison with commercial and research-based MAR algorithms

The performance of the developed Cycle-MAR network was compared with commercially available MAR algorithms and research-based MAR algorithms. iMAR and O-MAR were directly applied during the reconstruction of the phantom CT scans by the scanners. iMAR was not applied to the Siemens PET-CT scans, because it was not available on this particular scanner. In addition to this, MDT, CCS-MAR, and Cycle-MAR were also applied to all phantom and clinical CT scans.

### Image quality metrics analysis

Structural similarity (SSIM) index, root mean square error (RMSE) of the HU values, and peak signal-to-noise ratio (PSNR) [38, 55] were calculated to evaluate the performance of the Cycle-MAR network for metal artifact reduction and image quality improvement. These metrics were calculated for the CT_art_ and the CT_cor_ scans compared to the CT_ref_ scans. The analysis was performed using the overall mean values of these image quality metrics calculated for all CT_art_ and CT_cor_ scans.

### HU value restoration evaluation

For the clinical CT scans, HU value measurements on specific regions were performed on the CT_art_ scans, CT_cor_ scans and the CT_ref_ scan. The contour-based mean HU values and standard deviation (STD) were calculated for the entire heart, lungs, and bone regions from all CT slices using MatLab (The MathWorks Inc, USA) (See Fig. 5). The percentage of mean HU value improvement for CT_cor_ scans was calculated using the following equation,

1 $${\text{Mean}}\,{\text{HU}}\,{\text{value}}\,{\text{improvement}}\,\left( \% \right) = \frac{{\left| {\Delta HU} \right|_{{CT_{{ref,}} CT_{{art}} }} - \left| {\Delta HU} \right|_{{CT_{{ref,}} CT_{{cor}} }} }}{{\left| {\Delta HU} \right|_{{CT_{{ref,}} CT_{{art}} }} }} \times 100\%$$

where ${\left|{\Delta } \text{H}\text{U}\right|}_{{\text{C}\text{T}}_{\text{r}\text{e}\text{f}, }{\text{C}\text{T}}_{\text{a}\text{r}\text{t}}}$ and ${\left|{\Delta } \text{H}\text{U}\right|}_{{\text{C}\text{T}}_{\text{r}\text{e}\text{f}, }{\text{C}\text{T}}_{\text{c}\text{o}\text{r}}}$ indicate the absolute difference in the contour-based mean HU values between the CT_ref_ scans and the corresponding CT_art_ and CT_cor_ scans, respectively.

## Results

### Phantom scans analysis

**Fig. 4:**
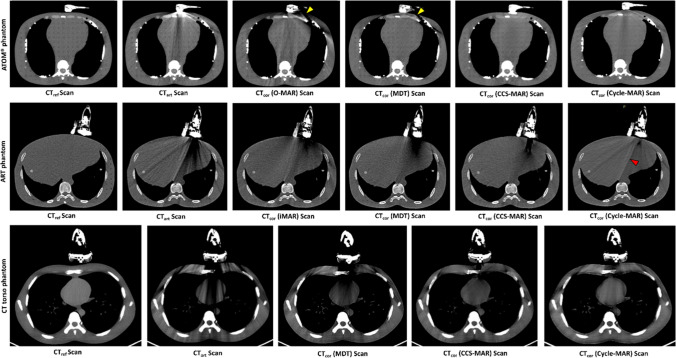
From top row to bottom: CT scans of the ATOM®, ART, and CT torso phantoms. The images from left to right show: the CT_ref_ scan with the US transducer details, CT_art_ scan, and the CT_cor_ scans after application of the MAR algorithms [Window level/width: 50/350]. The yellow marks indicate induced secondary artifacts, while the red mark indicates residual streaks artifacts, respectively

Figure 4 shows the CT_ref_ scans, and the corresponding CT_art_ and CT_cor_ scans from the ART, ATOM®, and CT torso phantoms. In general, Cycle-MAR outperformed other MAR algorithms to reduce the intense dark or bright regions near the US transducer during the visual inspection. Residual streak artifacts were observed on CT_cor_ after the Cycle-MAR application, especially in the ART phantom scans (red mark in Fig. 4). In the ATOM® phantom scans, O-MAR and MDT applications induced secondary dark streak artifacts (yellow marks in Fig. 4). The Cycle-MAR application on the CT_art_ scans generally improved the calculated SSIM and PSNR values, while the RMSE values were decreased (Table 3).

**Table 3:** Mean values of SSIM, PSNR and RMSE for the CT scans of the ATOM®, ART and CT torso phantoms

Image quality metrics	Phantom CT scans	CT_art_ scan	CT_cor_ Scans				
			iMAR	O-MAR	MDT	CCS-MAR	Cycle-MAR
Mean (± STD)							
**SSIM**	**ATOM®**	0.71 (± 0.12)	*N/A*	0.86 (± 0.05)	0.95 (± 0.07)	0.95 (± 0.08)	**0.95 (± 0.04)**
	**ART**	0.65 (± 0.19)	0.85 (± 0.08)	*N/A*	0.85 (± 0.04)	0.86 (± 0.06)	**0.87 (± 0.05)**
	**CT torso**	0.60 (± 0.27)	*N/A*	*N/A*	0.81 (± 0.14)	0.84 (± 0.17)	**0.88 (± 0.11)**
**PSNR (dB)**	**ATOM®**	19.02 (± 0.43)	*N/A*	29.02 (± 1.40)	31.03 (± 1.29)	31.38 (± 1.02)	**32.48 (± 0.26)**
	**ART**	16.41 (± 2.50)	24.99 (± 1.83)	*N/A*	25.11 (± 1.57)	**25.6 (± 1.65)**	25.58 (± 1.31)
	**CT torso**	18.56 (± 3.70)	*N/A*	*N/A*	26.31 (± 1.70)	26.75 (± 1.93)	**27.39 (± 1.10)**
**RMSE**	**ATOM®**	73.8 (± 9.90)	*N/A*	42.52 (± 5.83)	32.33 (± 6.24)	29.96 (± 4.84)	**28.11 (± 5.70)**
	**ART**	87.94 (± 6.72)	33.48 (± 3.36)	*N/A*	32.97 (± 3.38)	31.21 (± 4.21)	**30.59 (± 2.65)**
	**CT torso**	114.13 (± 9.71)	*N/A*	*N/A*	63.73 (± 5.43)	55.67 (± 4.43)	**53.33 (± 3.40)**

The best performance is indicated with bold numbers

*STD* Standard Deviation

### Clinical scans analysis

**Fig. 5:**
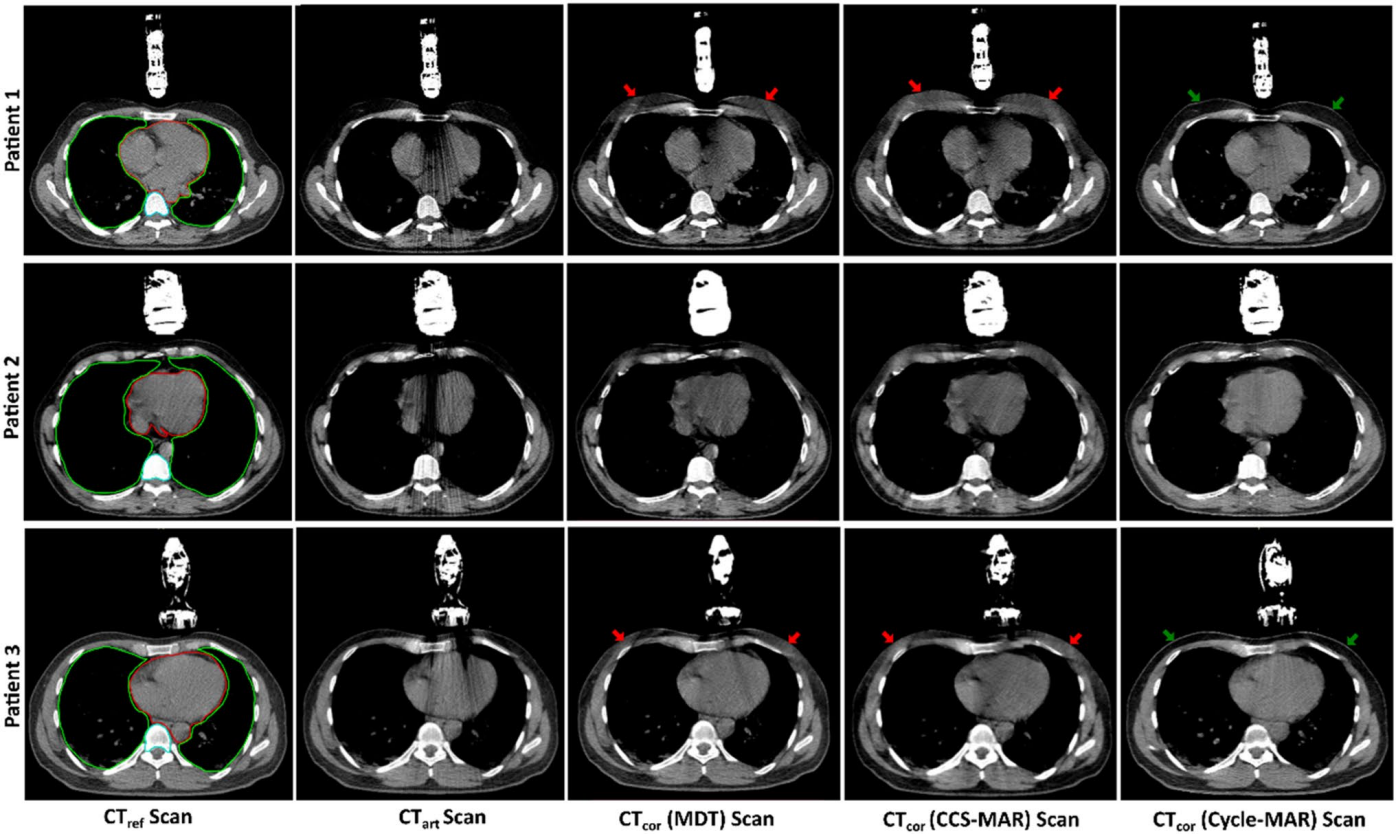
CT scans from three patients. The images from left to right show: the CT_ref_ scan with the US transducer details, CT_art_ scan, and the CT_cor_ scans after MDT, CCS-MAR, and Cycle-MAR applications [Window level/width: 50/350]. The red and green arrows indicate the changes of soft tissue density. The counters for the HU value measurements were shown on the CT_ref_ scans: the heart (red), bone (blue) and lungs (green)

An example of a CT_art_ scans from three randomly selected patients and the effect of MDT, CCS-MAR, and CycleGAN applications on them for metal artifact reduction is shown in Fig. 5. Based on the visual inspection, Cycle-MAR application on CT_art_ scans restored the soft tissue and bone details better than the MDT and CCS-MAR algorithms (red and green arrows in Fig. 5). The overall mean values of SSIM, PSNR, and RMSE for all clinical CT scans from 14 patients are shown in Table 4. Cycle-MAR application on CT_art_ scans generally resulted in higher mean SSIM and PSNR values and lower mean RMSE values.

**Table 4:** The overall calculated mean values of SSIM, PSNR and RMSE values for all clinical CT scans from 14 patients

Image quality metrics	CT_art_scan	CT_cor_ Scans		
		MDT	CCS-MAR	Cycle-MAR
Mean (± STD)				
**SSIM**	0.75 (± 0.21)	0.91 (± 0.08)	0.91 (± 0.09)	**0.92 (± 0.19)**
**PSNR (dB)**	17.57 (± 3.08)	25.86 (± 2.10)	25.21 (± 2.04)	**26.32 (± 1.80)**
**RMSE**	84.83 (± 11.25)	33.85 (± 10.05)	32.63 (± 9.09))	**30.86 (± 8.80)**

The best performance is indicated with bold numbers

*STD *Standard Deviation

Table 5 shows the overall mean (± STD) HU values for the CT_ref_ Scans, and the calculated absolute differences between the overall mean and the differences of standard deviation (STD) of HU values for the CT_art_ Scans, and CT_cor_ Scans compared to CT_ref_ scans for the heart, lungs, and bone regions on the clinical CT scans. The application of MAR algorithms improved the HU value measurements across all regions. Cycle-MAR application restored the mean HU values for the heart and lung region better than MDT and CCS-MAR.

**Fig. 6:**
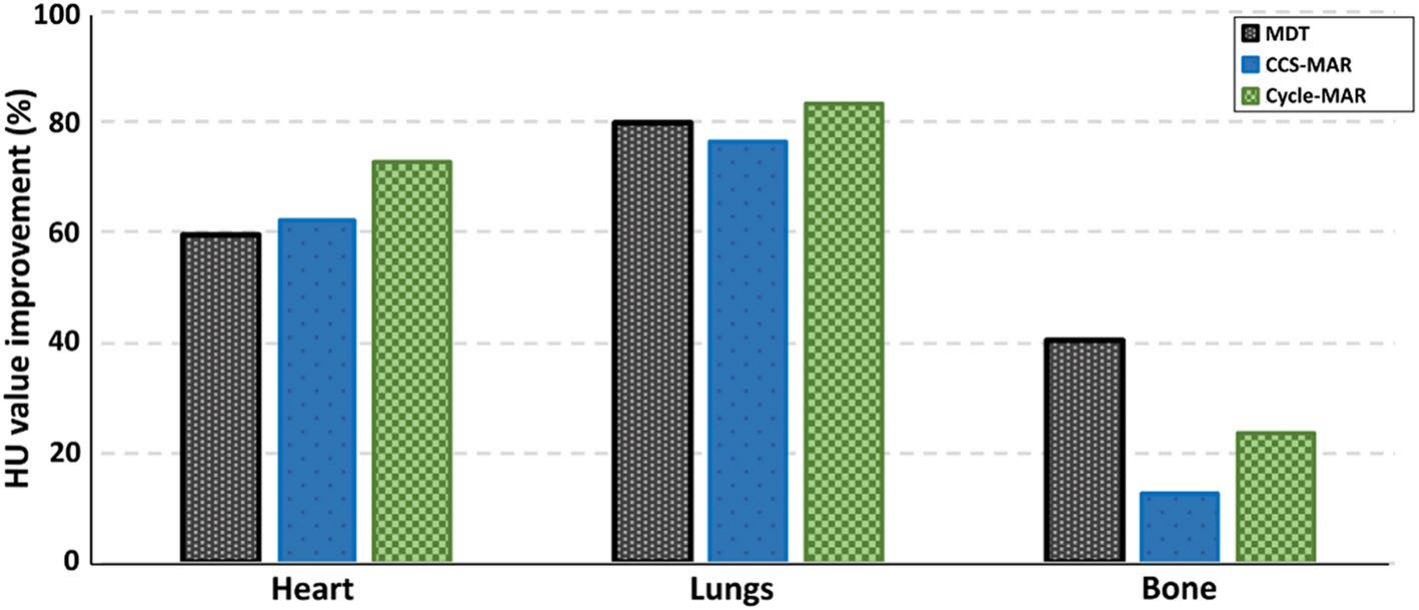
HU values improvement percentage for the heart, lungs and bone regions on the CT_cor_ scans after MDT, CCS-MAR and Cycle-MAR application

The percentage of mean HU value improvement for the heart, lungs and bone regions is shown in Fig. 6. For the heart region, in which typically the target is located, the improved HU value percentage after MDT, CCS-MAR, and Cycle-MAR applications was 59.58%, 62.22%, and 72.84%, respectively. The regions of lungs and bone were considered as OARs in this study, for these regions, the highest improvement percentage was found after the application of Cycle-MAR and MDT, respectively.

**Table 5:** The overall region-based Hounsfield unit (HU) value measurements and calculation using all clinical CT scans from 14 patients

Region	CT_ref_Scans	CT_art_Scans	CT_cor_Scans		
			MDT	CCS-MAR	Cycle-MAR
	Mean (± STD) HU	|Δ mean| (Δ STD) HU			
Heart	33.34 (± 37)	46.55 (15)	20.10 (3)	20.32 (2)	9.18 (7)
Lungs	− 675.00 (± 174)	30.01 (36)	6.00 (11)	7.01 (16)	5.00 (6)
Bone	233.00 (± 64)	40.69 (9)	29.00 (1)	35.53 (1)	31.04 (2)

|Δ mean| (± Δ STD) HU represents the absolute differences of overall mean and the differences of STD between CT_ref_Scans and the corresponding CTart _art_Scans, and as well as between CT_ref_ Scans and CT_cor_ Scans, respectively

*STD* Standard Deviation

## Discussion

In this study, a Cycle-MAR algorithm which used paired CT scans for training purposes was proposed to reduce the US transducer-induced metal artifacts on planning CT scans for US-guided cardiac radioablation. Cycle-MAR was evaluated for the improvement of image quality and HU value restoration compared to the commonly used commercial and research-based MAR algorithms.

Overall, the proposed model effectively reduced the metal artifacts on the clinical CT scans more than on the phantom CT scans. For the Cycle-MAR training, only clinical CT scans, and no phantom CT scans were used. This might be a reason for the noticeable residual streaks on the phantom CT_cor_ scans after the Cycle-MAR application (See Fig. 4). This potentially can be improved by adding separate sets of phantom scans to the algorithm training set. On the other hand, in the end, the algorithm will be used in the clinic and therefore good performance on phantom scans is less important. To further reduce the residual streaks, the CT_cor_ scans which resulted from the training of Cycle-MAR may be added to the training data set and this is under consideration for future work.

All MAR algorithms restored the measured HU values for the cardiac structures within 21 HU, which is well below the tolerance accuracy of 30 HU for the waterlike material recommended by the American Association of Physicists in Medicine (AAPM) guidelines [56] for image-guided radiation therapy. Remarkably, the same guidelines recommend that the HU value deviation for the lung and bone be within 50 HU.

The application of conventional MAR algorithms on CT_art_ scans modified the anatomical structures and induced a number of secondary artifacts (See Figs. 4 and 5). These MAR algorithms apply their correction on the projection data, therefore, small errors in local corrections in the projection data can affect the reconstructed CT scan globally [12]. However, Cycle-MAR works in the image space and does not require any projection data for artifact corrections. This means that the local changes are applied only to a specific area on the CT scan.

To the best of our knowledge, this is the first study to investigate the application of a deep learning model, CycleGAN, for the reduction of US transducer-induced metal artifact on CT scans which has been compared to state-of-the-art MAR algorithms. Even though Cycle-MAR generally well reduced the metal artifacts compared to other MAR algorithms, especially in the clinical CT scans, a reduction in image contrast was observed on the CT_cor_ scans after Cycle-MAR application. A possible reason for this is the inherent limitation of the generator in the conversion and/or reassignment accuracy of pixel values while performing feature extraction and the image translation process. In addition, the direct optimization in pixel differences through the loss function may also result in reduced image contrast or blurry appearance on CT scans [31, 57]. Therefore, investigating a different generator, especially DenseNet (Densely Connected Network) [58, 59] instead of ResNet, and also examining appropriate loss functions may solve this issue.

This work has a few limitations: the performance of Cycle-MAR was evaluated using clinical CT scans with the simulated US-transducer-induced metal artifacts. To draw final conclusions regarding the performance of the proposed MAR algorithm, further evaluation using clinical data with real artifacts is necessary. However, this can be a challenging task due to ethical justification of acquiring an additional CT scan [one with the probe in place (CT_art_) and another one without it (CT_ref_)].

In addition to the evaluation of image quality improvement and HU value restoration, the dosimetric impact of the metal artifact reduction including the accuracy in contouring, and the calculation of dose distribution for the arrhythmogenic tissue (target) and OARs during the treatment planning is also crucial. Future work will therefore include an evaluation of the dosimetric impacts of the application of the Cycle-MAR network.

## Conclusion

This work developed a MAR network based on a deep learning CycleGAN which can be used to reduce metal artifacts resulting from the presence of a US transducer during CT scan acquisition. The performance of the proposed algorithm was evaluated for the metal artifact reduction abilities on phantom and clinical CT scans in comparison with commonly used commercial and research-based MAR algorithms. The results of the study have shown that the proposed Cycle-MAR considerably reduces the metal artifacts, while preserving the bone density and soft tissue details. Future challenges and analysis include exploring appropriate loss functions for the improvement of adversarial training, and dosimetric evaluations using clinical CT scans.
